# Bioinformatics Projects Supporting Life-Sciences Learning in High Schools

**DOI:** 10.1371/journal.pcbi.1003404

**Published:** 2014-01-23

**Authors:** Isabel Marques, Paulo Almeida, Renato Alves, Maria João Dias, Ana Godinho, José B. Pereira-Leal

**Affiliations:** 1 Instituto Gulbenkian de Ciência, Oeiras, Portugal; 2 Escola Secundária Stuart de Carvalhais, Queluz, Portugal; Whitehead Institute, United States of America

## Abstract

The interdisciplinary nature of bioinformatics makes it an ideal framework to develop activities enabling enquiry-based learning. We describe here the development and implementation of a pilot project to use bioinformatics-based research activities in high schools, called “Bioinformatics@school.” It includes web-based research projects that students can pursue alone or under teacher supervision and a teacher training program. The project is organized so as to enable discussion of key results between students and teachers. After successful trials in two high schools, as measured by questionnaires, interviews, and assessment of knowledge acquisition, the project is expanding by the action of the teachers involved, who are helping us develop more content and are recruiting more teachers and schools.

## Background and Motivation

Our lives are increasingly touched by science and technology, from the everyday activities of browsing the internet, taking a prescription drug, etc., to major societal discussions involving, for example, genetically modified foods, cloning, or stem cells. It is therefore imperative that we engage young people in science. We witnessed in the past shrinking numbers of students choosing science degrees for their university education [1]. This trend seems, however, to have been inverted both in Europe and in the United States [2], [3]. A recent study points to the development of new and more attractive curricula and teaching methods as the driver for this increased interest [3]. In light of the growing evidence of a direct link between attitudes towards science and the way science is taught [1], there is increasing recognition of the need to couple the traditional teacher-centred “deductive approach” to the learner-centred “inductive approach,” relying on observation, experimentation, and teacher guidance in constructing students' knowledge. This “bottom-up” approach, called enquiry-based learning (also known as problem-based learning or case-based learning) [4] recapitulates the scientific process (raising questions, collecting data, reasoning, reviewing evidence, drawing conclusions, and discussing results), thus promoting both ideas *of* science (scientific concepts) and ideas *about* science (process, practices, and critical thinking), i.e., about the Nature of Science (NOS).

Bioinformatics is a discipline at the intersection of biology, computer science, information science, mathematics, and to some extent also of chemistry and physics. It developed in response to the increasingly complex data types and relationships in biological research, addressing the need to manage and interpret biological information. This interdisciplinary nature makes bioinformatics an ideal framework to engage high school students, as it illustrates the interplay between different scientific areas, while touching on many aspects that are relevant to the younger generations—health, environment, etc. This has been recognized by many others who have implemented bioinformatics-training programs. Examples are a web-based, problem-oriented approach aimed at introducing students to bioinformatics [5] and the use of bioinformatics activities as a way to teach evolution [6] or notions of polymorphisms in the context of human genetic variation and disease [7]. Bioinformatics has also integrated with wet-lab activities in initiatives like the student-aimed “Cus-Mi-Bio” project [8], which include gene finding activities, or in projects aimed at high school and college teachers, such as the ones at the Dolan DNA learning centre of Cold Spring Harbor Laboratory involving plant genome annotation [9]. More recently, activities that aim to introduce high school students to bioinformatics itself have also been reported [10], and, as of 2012, an exercise using Basic Local Alignment Search Tool (BLAST) has been included on the Advanced Placement, high school biology, national test in the US (http://apcentral.collegeboard.com/apc/members/courses/teachers_corner/218954.html). Note, however, that these are likely isolated cases rather than the norm, as a survey revealed that in 2008 bioinformatics was still absent from the classroom in the US [11], and likely elsewhere.

## The “Bioinformatics@school” Program

We run a Bioinformatics Core at the Instituto Gulbenkian de Ciência, in Portugal, that has long been engaged in outreach activities. In 2007, we decided to implement a genomics/bioinformatics activity that would enable enquiry-based learning; link to the national curricula in biology in secondary education; introduce students to bioinformatics, genomics, and molecular biology, areas that underlie many of the key debates and products in our societies; foster active learning, making use of technologies that younger generations are increasingly comfortable with; and help teachers incorporate the latest advances in science into their teaching. We developed a prototype system that we describe the following components of here: its development, implementation, and the results of nearly five years of activity.

**Figure 1:**
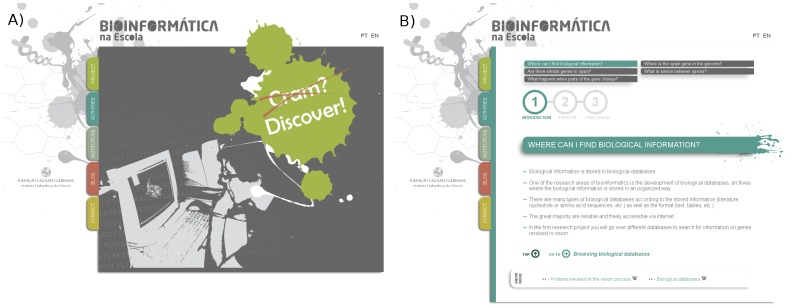
The Bioinformatics@school web portal. (**A**) Screenshots of the home page. (**B**) Screenshots of exercises pages.

We developed and implemented a framework for the use of bioinformatics-based research projects in high schools to support the life-sciences curricula, which we named, in Portuguese, “Bioinformática na escola,” loosely translating to “Bioinformatics@school.” It consists of research projects that may be conducted independently by high school students of different ages, either under direct teacher supervision or as homework. Each work unit in a research project is designed to be carried out in 90 minutes, which is a standard class length in Portuguese high schools. We implemented it as a web portal (Figure 1A, 1B)—www.bioinformatica-na-escola.org. Although primarily written in Portuguese, the site makes use of external, freely accessible bioinformatics tools and databases available in English. This is not a problem for Portuguese high school students that typically start learning English at the age of nine. Because of the dependency on external sites, we have ensured that students are given alternative access to any data on which progression to the following activity depends.

The whole program is structured as a set of *projects* with open-ended questions. A project may have a single *activity* or several, each having focused questions. Answering these focused questions enables students to discuss and/or solve the project's main question. Individual activities in the multi-activity projects were designed to also be used independently (discussed below). The concept lends itself both to classroom use, individually or in pairs, or as homework. We designed individual activities to explore specific concepts that are part of the school curriculum and the projects to be coherent with the curriculum of specific age groups, with the active collaboration of teachers in choosing the topics.

Projects are organized as follows. Once a project is selected, the student has access to a page that summarizes the problem to be solved and a link to the first activity. As the student enters one activity, s/he is presented with a sequential series of pages, each giving some background information on the specific problem the student has to follow and a brief description of the bioinformatics resources/tools to be used. At the end of each activity, the student is taken to a summary page (“now you know that…”) with an overview of the basic concepts that were addressed in the activity. All pages include links to additional information on key concepts, mostly on Wikipedia (www.wikipedia.org), including explanations about the resources and algorithms used in the analysis. Once the activity/final activity is complete, the student is taken to a summary page that reviews the key concepts of the project as a whole and a series of questions that act as primers for discussion amongst students and with the teacher(s) (see Figure 1A, 1B for screenshots). Table 1 summarizes the questions, concepts, and software and resources that are covered in each individual activity of “Vision,” the first multi-activity project that we have implemented in the Bioinformatics@school portal (further detailed in Text S1). Its implementation in schools is discussed below.

**Table 1:** Individual activities in the project “Vision.”

#	Concepts	Question	Software/Databases
1	Biological databases, accession numbers/identifiers	Where can I find biological information?	GeneCards, UniProt/Swiss-Prot, PubMed, OMIM, Human Gene Mutation Database
2	Gene structure, sequence motifs, gene finding	Where is the ‘opsin’ gene in the genome?	GeneMark, WWWPromoterScan
3	Genetic code, ORFs, homology	Are there similar genes to opsin?	NCBI's ORF finder, BLAST
4	Protein structure (2^ry^ and 3^ry^), structural coordinate files (.pdb)	What is similar between opsins and related proteins?	Clustalw, PDB site, Rasmol
5	Genetic variation (mutation, SNP), karyotype, allelic frequencies, homo/heterozygosity	What happens when parts of the gene change?	Ensembl, OMIM

The global aim of the project is that students discuss how we see in colour, how this is genetically encoded, that there are conditions where colour vision is lost, and that not all animals see the same colours. The project is divided into five individual activities, each with specific questions, that collectively give students the ability to discuss the overarching problem.

## Implementing “Bioinformatics@school”

### Iterative development of project modules

We started to develop “Bioinformatics@school” as a pilot project in 2007, in close collaboration with high school students and teachers. The first stage of the project consisted of identifying the topics within the high school curricula that would be amenable to bioinformatics treatment, as well as the ideal school year for the pilot to be developed. We chose 12th grade biology, in the last year of high school in the Portuguese educational system, as their curricula included multiple themes that were ideal to address using bioinformatics (such as genes, genomes, genetics, evolution, mutation, etc.), and these students would all have had several years of English language schooling (discussed above). The next phase of the project consisted in the enrollment of schools. Two secondary schools located in the Lisbon area were recruited, representing two different demographics. Escola Secundária Miguel Torga (ESMT), in Queluz, is a large suburban school that covers a variety of social strata, while Escola Secundária Quinta do Marquês (ESQM), in Oeiras, is located in a high income area with high levels of graduates and post-graduates. We engaged seven 12th grade Biology teachers, two from ESMQ and five from ESMT. One hundred and fifty students were involved in this initial pilot phase, representing multiple science-related career ambitions, ranging from engineering, health, biology, psychology, sports, etc.

We conceived the general framework described above and developed the first full project consisting of five activities, aimed at understanding how animals see different colours (“Vision” project, Table 1). We chose this question because we believed it to be sufficiently intriguing and relevant to engage the students (natural variants cause differential colour perception between species and between different people), but also for practical reasons—the biology of light detection via opsins is well understood, as is the 3D structure of opsins. The aim of the project is to motivate a discussion about evolution, molecular mechanisms, and disease, all inferred from bioinformatics analysis, while helping teachers and students engage with specific topics of the Life Sciences curriculum via the individual bioinformatics activities.

An innovative aspect of this project was the collaboration between scientists, teachers, and students on different aspects of the development, implementation, and testing—a three-way dialogue with continual updating in response to feedback of students and teachers. The development was iterative, first within our Bioinformatics Unit, and then in discussions with teachers. Once a first prototype was in place, one of us (IM) went to the schools to guide the students in the first activities of the project, with the help of the teacher. Student feedback was then used to improve the activities, in terms of rationale, language, and presentation.

### Teacher training

Keeping up to date with the rapid developments in genomics and bioinformatics represents a challenge for high school teachers, particularly when many may have completed their training decades ago. In fact, in our experience, bioinformatics is a novel subject area to most Portuguese high school teachers. This led us to implement a parallel teacher-training program, again co-developed with the first set of teachers. Teachers were trained by bioinformatics experts, with the main goal of training to guide the students in the bioinformatics-based projects and to understand the basics of the bioinformatics methods and resources underlying each activity. We developed a teacher's manual that described the activities step by step and provided additional background information for the teacher to be comfortable with all the concepts in each activity. The teacher training consisted of having the teachers follow the same activities as the students, with the help of the teacher manual and under the supervision of a bioinformatician. We have expanded the teacher training to include seminars about applications of bioinformatics to human health, biotechnology, etc. A typical teacher training program lasts about 25 hours.

## Extending the Program and Sustainability

**Figure 2:**
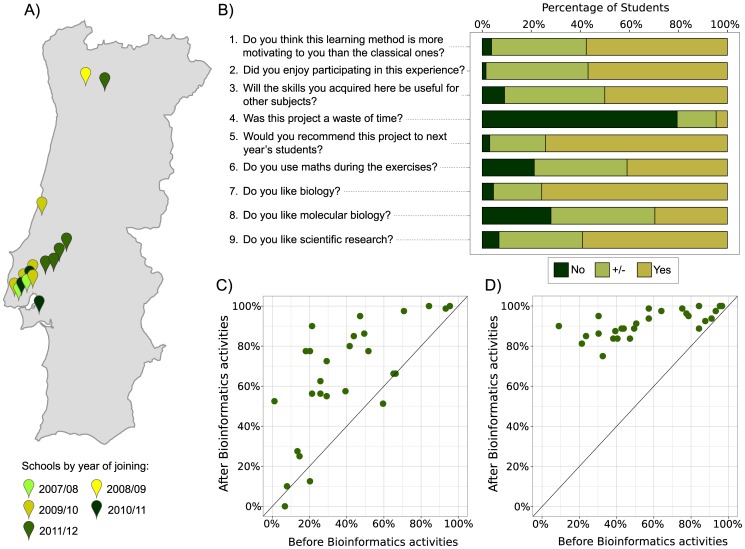
Schools and evaluation. (**A**) Map of schools participating, coloured by year of joining the project. (**B**) Summary of responses to confidential questionnaire. (**C**) Knowledge acquisition—each dot represents one class and the average score that students in that class achieved in the test before and after finishing the “Vision” project. (**D**) Confidence—each dot represents one class and the percent of answers that students in that class answered True or False, as opposed to answering “I don't know,” before and after finishing the “Vision” project.

After the successful pilot stage in 2007 the project has expanded to other geographical areas of Portugal. Thirty three new schools have joined the program, some via previously engaged teachers who took the program with them when they moved to a new school, others by new teachers who contacted us, after hearing about the project, and asked us to help them implement it in their schools. In total, schools of 11 municipalities in four Portuguese districts are currently following the program (Figure 2A). On their own initiative, some teachers have adapted the individual activities within the “Vision” project for use with younger students. They have also picked individual or subsets of activities and re-used them with different genes/systems, combining them in novel ways, to create new projects. They have also engaged with us to develop new projects (“Tasting Bitter”) and activities (“Tree of Life”). Furthermore, teachers are recruiting and training new teachers to use our activities. Interestingly, we observed that teachers tailored the activities to their own teaching style, some engaging the students almost at every mouse click, whereas others would only focus on explaining the basic ideas at the beginning and then discussing the outcomes at the end.

One aspect that worried us from early on was how to motivate teachers to engage with projects like ours when they are overwhelmed with teaching and administrative work. We realized that certification of the training is important for career progression within the Portuguese public educational system. We invested in having the project certified for teachers' continuous professional development by the national educational authorities (Conselho Científico-Pedagógico da Formação Contínua), thus making engagement with Bioinformatics@school even more appealing to the teachers. Recently we established a partnership with a teacher training centre (Centro de Formação Lezíria - Oeste) to enable other teachers in another Portuguese region to receive training in Bioinformatics activities and further promote the decentralization of “Bioinformatics@school.”

We have, thus, reason to believe that the use of the Bioinformatics@school platform is spreading on its own, with a dynamic beyond the ability of the small staff at the Bioinformatics Core that developed it.

## Impact Assessment

We wished to evaluate how students and teachers perceive the program and to what extent it is an effective learning tool. These are independent questions that we addressed using different approaches. Conversations with students participating in the program suggested that they were motivated to participate in “hands-on” activities We implemented a simple confidential questionnaire to capture students' views beyond anecdotal opinions, that was given to 150 students (two schools, seven classes), during the implementation phase of the project. The results are shown in Figure 2B and reveal that the majority of the students found the approach used in this project more motivating than traditional teaching methods (58%), and enjoyed participating in it (60%). About 80% considered it had not been a waste of time and 80% would recommend the project to next year's colleagues. This type of questionnaire is useful in gauging attitudes towards the program, but it has caveats, namely that the students at this stage were very involved with the development of the Bioinformatics@school project and may be overly positive because of that. In addition, it gives no information about student learning. To address this, we devised a simple test on the concepts explored in the program, with “True/False/I Don't Know” answers (Table S1). We asked four classrooms to take the test before and after the activities (this test was irrelevant for their grades). Plotting the percentage of correct answers per student before and after the activities (Figure 2C) revealed a dramatic increase in the proportion of correct answers, indicating that students actually gain knowledge. One surprising result was that the students appeared more confident after doing the activities: they increasingly answered the test questions as false or true, rarely using “I don't know” (Figure 2D). Since most of the concepts in our activities are part of the school curricula and were being covered in class by their teachers, we speculate that the decrease in “I don't know” answers may indicate that students are less afraid of venturing answers to scientific questions after doing the activities. Fear of science (“too complicated”) has been pointed out as a reason for the decreasing number of students pursuing scientific degrees [1]. This is an exciting finding that we will need to specifically evaluate further in the future. Regarding the teachers, we developed the whole program in close collaboration with them and obtained continuous feedback on the content and presentation. Although we have not as yet conducted a systematic evaluation of teachers' views about the program, the continuous contact with the currently more than seventy teachers involved suggests to us that this is a useful teaching/learning tool. In particular, teachers mention that these activities allow them to overcome the lack of laboratory-based practicals associated with some of the content in the curricula, like genetics and molecular biology. The fact that the program is spreading, with new teachers and schools recruited by word of mouth by the teachers themselves, underscores its interest and usefulness to teachers.

## Discussion and Future Directions

In summary, we implemented a set of bioinformatics multi-activity research projects designed to enable enquiry-based learning in high schools. Assessment of this project has shown that students find it enjoyable and teachers believe it to be useful as a teaching aid. Objective assessment of knowledge acquisition revealed a clear positive effect both in knowledge and confidence of the students. Teachers have taken the initiative to adapt the activities to their own teaching settings and are also recruiting other teachers, which gives us further confidence in the usefulness of this project.

We have focused the projects on addressing specific biological questions, to serve the Life Sciences curriculum. This means that we don't explore the algorithmic or technological side of bioinformatics. For the future, we hope to engage teachers from mathematics, information technology, physics, and chemistry to develop projects that can serve the curricula of those particular subjects.

Recently, Form and Lewitter proposed a simple set of ten rules to guide the use of bioinformatics in high schools [12]. While these were not available at the time we were developing this project, it is interesting to note that we independently “discovered” several of these principles. We implemented individual activities with clear, simple goals (rule 1) that built on each other (rule 4), enabling students to “discover” concepts on their own (rules 5 and 8). Throughout this project we were always mindful that these activities need to serve the pre-existing curricula (Rule 3). In the future we would like to have multiple projects serving the same concepts that would allow students in each class to choose an individual project (rule 6: personalization) that they could then present and contrast to other projects pursued by their colleagues (rule 10: produce a product). We would like to develop a mapping of activities to concepts in the curricula, so that it becomes even easier for teachers to mix and match the individual activities to different contexts, thus using our project as a means to empower the teachers. Based on our experience in setting up this program, we would like to suggest two additional “simple rules” that we believe to be important when developing contents to be used in high schools:

1. Engage teachers and students in the development of the activities, as a means of empowering them and ensuring that the end product meets all the cognitive and pedagogical requirements (e.g., engage the teachers in choosing the specific topics of the curricula that would benefit from bioinformatics-based projects as well as to advise on time or practical constraints on their use in the school setting; engage both teachers and students to identify weak/unappealing points in the contents and formats of the activities and to suggest better solutions, etc.).
2. Evaluate the impact of the activities on engagement/enthusiasm for science and, in particular, on knowledge acquisition, as demonstrated effectiveness is the best way to get bioinformatics into the classroom. In our opinion, perpetuating useless activities just for the sake of their perceived modernity is more likely to harm the use of bioinformatics as a tool for high school science education than to advance it.

Our program was developed in Portuguese as it is targeted at Portuguese students. While this gives us potential access to a universe of more than 200 million Portuguese speakers worldwide, it is hard to use by speakers of other languages. We have started translating the whole set of activities into English, thus making Bioinformatics@school accessible to a much larger target audience. Equally, besides developing novel activities, we would like to adapt those from successful experiments elsewhere, and in due time will contact their authors directly. In this regard, the existence of a central repository of bioinformatics exercises to be used in high schools, with clear explanations according to pre-defined standards and mapping to specific concepts, would facilitate the adoption of bioinformatics in high schools. Developing standards and repositories should come naturally to the bioinformatics community!

## Supporting Information

Table S1 Questionnaire for impact assessment.
(DOC)

Text S1 Activities in the “Vision” project.
(PDF)
