# Supplementary material for: Bioinformatics Projects Supporting Life-Sciences Learning in High Schools
Source: PLoS Comput Biol. 2014 Jan 23;10(1):e1003404. doi: 10.1371/journal.pcbi.1003404 (PMC3900377; doi:10.1371/journal.pcbi.1003404)
Supplement: Table S1 — Questionnaire for impact assessment. (DOC) [file pcbi.1003404.s001.doc]

## Bioinformatics @ School - Questionnaire (figure 2C,D)

Please fill in this questionnaire. Your opinion is of great importance to evaluate the project ‘Bioinformatics at School’.

According to your knowledge, please answer the following statements by marking your answer with a cross (X):

Gender ______

|  | Questions | True | False | Don't Know |
| --- | --- | --- | --- | --- |
| 1 | Mathematics, statistics, computing etc. are indispensable to the study of biology. |  |  |  |
| 2 | There are specific databases to store biological information. |  |  |  |
| 3 | The complete sequence of the human genome is still unknown. |  |  |  |
| 4 | The human genome is made up of about three billion base pairs |  |  |  |
| 5 | The human genome contains more than 100 thousand genes. |  |  |  |
| 6 | Computational methods can be used to identify genes in genomic sequences. |  |  |  |
| 7 | In the human genome it is possible to visualize the structural organization of already known genes. |  |  |  |
| 8 | Splicing is the processing of proteins. |  |  |  |
| 9 | One gene only produces one transcript. |  |  |  |
| 11 | The genomic DNA transcription begins immediately after the TATA box signal |  |  |  |
| 12 | In the process of translation, the start codon is not always in the beginning of the messenger RNA. |  |  |  |
| 13 | UTRs are untranslated regions of messenger RNA. |  |  |  |
| 14 | Homologous proteins are defined by not having a common ancestor. |  |  |  |
| 15 | We infer homology between two or more proteins by detecting similar regions in the amino acid sequences when aligned. |  |  |  |
| 16 | A great similarity between two proteins indicates, in general, they have the same function. |  |  |  |
| 17 | Most of the tertiary structures of proteins were determined experimentally by X-ray crystallography or Nuclear Magnetic Resonance Spectroscopy (NMR). |  |  |  |
| 18 | The 3D structure prediction of proteins, from the sequence of their amino acids, is made with greater confidence by computational methods. |  |  |  |
| 19 | Nonsynonymous mutations never change the 3D structure of proteins. |  |  |  |
| 20 | Insertion and deletion mutations occur only at one nucleotide level. |  |  |  |
| 21 | When single nucleotide variations occur in at least 1% of the population, they are considered polymorphisms or SNPs (Single Nucleotide Polymorphism). |  |  |  |
| 22 | The International 1000 Genomes Project aimed to identify the most detailed map of human genetic variation. |  |  |  |
| 23 | Approximately 80% of the human genome is common to all people. |  |  |  |
| 24 | It is estimated that the genome of each person has about 100 SNPs in every 1200 nucleotides. |  |  |  |
| 25 | Most of the SNPs predicted for the human genome are not functionally relevant, i.e., they do not alter the function of proteins. |  |  |  |
| 26 | 80% of all SNPs seem to be important in the study of disease or have a role in pharmacogenomics. |  |  |  |
| 27 | The mapping of SNPs allows the identification of genes associated with various diseases such as diabetes, cancer, cardiovascular diseases, neurological diseases and alcoholism. |  |  |  |
| 28 | In the future, two people with the same disease will be treated with different drugs. |  |  |  |

Thank you very much for your participation

Bioinformatics Unit

Instituto Gulbenkian de Ciência

&

Secondary High School________________________________
