# Supplementary material for: Bioinformatics Projects Supporting Life-Sciences Learning in High Schools
Source: PLoS Comput Biol. 2014 Jan 23;10(1):e1003404. doi: 10.1371/journal.pcbi.1003404 (PMC3900377; doi:10.1371/journal.pcbi.1003404)
Supplement: Text S1 — Activities in the “Vision” project. (PDF) [file pcbi.1003404.s002.pdf]

Bioinformatics@School - 'Vision' Project'

This research project is composed of five activities, each one comprising a short introduction, research pages and conclusion. The activities are independent, ranging five different but interconnected exercises on different biological concepts:

Activity 1 - Where can I find biological information?

The main objective of the first activity is to instruct students on how to browse databases by giving them a few concepts on accession numbers/identifiers and the specificity of different databases such as UniProt/Swiss-Prot, PubMed, OMIM and HGMD. After reading a short introduction on biological databases, students follow a research page to browse the GeneCards database, using *opsin* as keyword. On this activity students learn about the gene responsible for capture of red color in vision by following links to other databases. Among the resources they will be presented with information about the genomic location of the gene, the protein for which this gene codes for, PubMed publications on this subject, which other animals have similar proteins, which mutations are known for this gene and what happens when parts of the gene change. At the end of this activity students should know that there are many different databases and they should be able to make conclusions on their own about content specificity and what kind of information they can retrieve from them.

Activity 2 – How is the opsin gene organized?

The activity starts with a short description of the Human Genome Project and the Entrez, UCSC and Ensembl databases used to explore genomes. Footnotes exist on most pages where students can find links to additional information about the subject. The aim of this activity is to find a coding region and the respective promoter region on a nucleotide sequence using bioinformatics online resources. On the first part of the research page they use the gene finding software GeneMark to predict a gene: a coding region, based on start and stop codons and splicing signals. On the second part they use the Promoter Scan software to discover motifs upstream of the coding region. At the end of the activity students should understand how genes are organized: exons and introns, 5´ and 3´UTRs, PolyAs, the

core promoter TATA box and TSS. It is recommended that students draw a picture and identify the positions of all the markers they found in the nucleotide sequence.

Activity 3: Are there similar genes to opsin?

This activity intends to introduce students to the concept of homology and protein families. After a short introduction on homology and the BLAST program they follow a two step research page. On the first part students use NCBI´s ORF Finder to translate a nucleotide sequence, the putative gene they found in the Activity 2. Here they get informed on the genetic code and the possible six frames of reading a nucleotide sequence. On the second part they learn the concepts of identity, similarity and the significance of E-value by using NCBI´s BLASTP software with the previously translated sequence as query. At the end of the activity students should know that the translated sequence is already deposited in a database and that there are other human opsin proteins very similar to the red one. Comparing the E-values obtained on BLASTP students should conclude that human opsins are homologs and part of the same protein family.

Activity 4: What is similar between opsins?

The aim of this activity is to introduce students to the relation between protein function and its structure. In this activity students follow a research page with instructions to align a list of amino acid sequences of human opsins using the ClustalW application from EBI. Here they are presented with a Venn diagram of amino acid properties and are capable of identifying the conserved regions on the alignment. On a second part of this research page they understand the importance of conserved regions by following a 3D visualization of a very well known opsin - bovine rhodopsin molecule. RasMol was the selected program because it's free, user friendly and simple to install. During this exercise students learn about the different levels of protein structures and methods to study 3D structure of proteins. At the end of this activity they should know that the chromophore, the receptor of light, binds to one specific amino acid which is common to all opsins. They should also know that there is one other group of amino acids, highly conserved, that is involved in the transmission of the light signal and a third group which varies between red, green and blue opsins and is structurally involved in the absorption of the wavelengths of the respective opsins. They should also be

introduced to the idea that modifications in any of these groups can potentially alter the function of the protein.

Activity 5: What happens when parts of the gene change?

This activity intends to explain students what is a mutation, different types of mutations and their consequences. They also learn what a SNP is and are informed about the 1000 Genome Project. To learn more about genetic variability they browse Ensembl using the OPN1LW human gene, responsible for red light capture. Following the research page, students browse chromosome X, look for the OPN1LW gene, and see how SNPs are distributed along the different regions of the gene: UTRs, introns, exons and promoter regions. After answering a few questions related with a specific SNP, students search for the respective allelic frequency on different populations and gender. At the end of the activity they should know that mutations are considered SNPs when they are present in more than 1% of the population; SNPs are responsible for the genetic variability of the human genome and, although they are of extreme importance to identify disease-associated genes, not all these variations are relevant for genetic studies.

Final Conclusions

The completion of this activity allows students to gain insight into Molecular Biology using Bioinformatics methodologies. Starting with a fragment of human DNA and searching for available biological information, students are able to identify: a gene (Activity 1 and 2), the correspondent protein (Activity 3), homolog proteins (Activity 3), secondary and tertiary structure of the protein (Activity 4) and mutations affecting protein function (Activity 5). On Activity 1 they also learn that all biological information can be stored in databases. Parallel to the execution of these activities students had access to information on genes involved in vision. With the knowledge gained, they should have no difficulty in answering the questions initially proposed for the Vision Project: What molecules are involved in the mechanism of vision? Why do we see different colors? What happens when these molecules change? Why don´t all organisms see the same colors? To answer this last question the teacher's manual advises to draw students'

attention to the fact that there are more than one type of photoreceptor cells and that not all animals have the same type and quantity of these cells.
